# Impact of a glutamine-enriched peptide formula on gastrointestinal toxicity and on the interruption of oncologic treatment in patients with adenocarcinoma of the rectum

**DOI:** 10.3389/fnut.2024.1414367

**Published:** 2024-12-20

**Authors:** Bárbara Gabriela Salas-Salas, Laura Ferrera-Alayón, Alicia Calleja-Fernández, Rodolfo Chicas-Sett, Eva Nogués-Ramia, Juan Zafra-Martín, Marta Lloret

**Affiliations:** ^1^Department of Radiation Oncology, University Hospital of Gran Canaria Dr. Negrín, Las Palmas de Gran Canaria, Spain; ^2^University of Las Palmas de Gran Canaria, Las Palmas de Gran Canaria, Spain; ^3^Department of Medical, Adventia Pharma, Las Palmas de Gran Canaria, Spain; ^4^Department of Radiation Oncology, Ascires Grupo Biomédico, Valencia, Spain; ^5^Department of General Surgery, University Hospital of Gran Canaria Dr. Negrín, Las Palmas de Gran Canaria, Spain; ^6^Department of Radiation Oncology, Hospital Universitario Virgen de la Victoria, Málaga, Spain; ^7^Department of Oncology University Hospital San Roque, Canarian Comprehensive Cancer Center, Las Palmas de Gran Canaria, Spain

**Keywords:** peptide diet, oral nutritional supplement, radiotherapy, rectal cancer, glutamine

## Abstract

**Background:**

Patients with rectal cancer may develop gastrointestinal toxicity associated with chemo-radiotherapeutic treatment that conditions their clinical, functional, and nutritional evolution. The aim of the study was to evaluate the efficacy of nutritional supplementation with a glutamine-enriched peptide diet (PD) compared to exclusive dietary advice (DA) on gastrointestinal toxicity, interruption of oncologic treatment, and nutritional evolution in patients with rectal cancer undergoing neoadjuvant treatment.

**Methods:**

Prospective cohort study with two groups. Patients with rectal cancer in treatment with neoadjuvant chemo-radiotherapy were recruited. One group of patients received nutritional supplementation with PD, and another group received DA exclusively, from the beginning of radiotherapy until the time of surgery. Intestinal toxicity was evaluated with the CTCAE 5.0 scale, functionality with the ECOG scale and nutritional status with GLIM criteria.

**Results:**

Fifty-four patients were initially selected, although 51 were finally enrolled: 25 in the PD group and 26 in the DA group. There was a reduction in the risk of diarrhea in the PD group midway through radiotherapy treatment [RR of 0.218 (95% CI = 0.052–0.923)] and at the end of treatment [RR of 0.103 (95% CI = 0.020–0.537)], as well as a reduction in the risk of developing mucositis at the end of treatment [RR of 0.405 (95% CI = 0.280–0.584)]. The use of a PD also decreased treatment interruptions with radiotherapy in stage III patients (0 vs. 15.8%, *p* = 0.049) and in malnourished patients (0 vs. 18.2%, *p* = 0.040).

**Conclusion:**

The glutamine-enriched peptide diet had a protective effect on the development of diarrhea and mucositis associated with chemo-radiotherapeutic treatment in patients with colorectal cancer under neoadjuvant treatment, as well as the interruption of radiotherapeutic treatment.

## 1 Introduction

Recent years have seen great advances in the treatment of oncology patients. Still, toxicity related to radiotherapy and chemotherapy treatment, or the combination of both, remains high. Cancer patients who undergo this type of therapy often present with symptoms that severely impair their clinical, functional, and nutritional outcome. Specifically, radiotherapy to the pelvic region has been found to be a main cause of nutritional deterioration, mainly due to radicular enteritis, which causes diarrhea, mucositis, abdominal pain, and, to a lesser extent, constipation (1).

Diarrhea related to cancer treatment (DRTO) is a side effect that causes deterioration of the patient’s nutritional status, treatment interruptions, frequent hospitalizations, and impairment of quality of life (2, 3). The prevalence of DRTO can reach up to 74% of cancer patients, depending on radiation doses, cancer treatment, female sex, low BMI, advanced age, and having undergone abdominal surgery (3). It is essential to treat DRTO early and perform the most appropriate intervention to minimize its progression to more severe states that could condition the continuity of cancer treatment and its survival (4). In clinical practice, early and precise nutritional intervention can favor the control of diarrhea, cover nutritional needs, and promote good nutritional status (5).

Cancer patients frequently present with a high risk of malnutrition *per se* due to the tumor itself, its location and extension, the oncologic treatment received (surgery, radiotherapy, chemotherapy), the toxicity related to it, the metabolic changes that develop, and their social environment (6). Previous studies have shown that malnutrition leads to a higher rate of hospital admissions, longer hospital stays, a lower quality of life, and higher mortality related to a decrease in the tolerance of oncologic treatments (7). Considering the negative effects of malnutrition in cancer patients, it is essential to detect it early and provide optimal nutritional support to minimize its progression.

Given the high prevalence of DRTO and malnutrition in the cancer patient, it is striking that clinical practice guidelines focus their recommendations on the pharmacological treatment of diarrhea but do not specifically address the nutritional support needed by patients (3, 8–10). The nutritional support plan will range from dietary advice (DA) to the use of commercial formulations, including oral nutritional supplements, enteral tube feeding, or even parenteral nutrition, depending on the severity and persistence of symptoms (11). Oral nutritional supplements may prove the most common and effective tool to treat both symptoms, as long as adequate adherence to treatment is achieved (12).

A peptide diet (PD) may be a nutritional therapy option for patients with DRTO due to its ease of absorption, suppression of pro-inflammatory cytokine production, and maintenance of mucosal integrity (13–15). There are few published studies, however, on the efficacy of nutrition with intestinal peptides in patients with diarrhea associated specifically with colorectal cancer therapy, although there are studies with enteral supplementation with glutamine that show positive results in improving the severity and symptomatology of patients with radicular enteritis (16).

The main studies on PD published to date have been conducted in cancer patients undergoing chemo-radiotherapy treatment but at the level of the oral mucosa, esophagus, stomach, or pancreas, showing heterogeneous results (17–24). For all these reasons, Sanz-Paris et al. (25) published an algorithm on the nutritional management of DRTO from an oligomeric formula. Based on this algorithm, these authors presented results on the clinical and nutritional efficacy of the implementation of this protocol in clinical practice, with very promising results (26). In 2023, Peña Vivas et al. (27) published a clinical study demonstrating that supplementation with PD reduces DRTO with respect to a polymeric diet, affecting the functional and nutritional improvement of the patient with rectal cancer in neoadyuvancy.

The aim of the study was to evaluate the efficacy of nutritional supplementation with a glutamine-enriched peptide diet (PD) compared to exclusive dietary advice (DA) on gastrointestinal toxicity, interruption of radiotherapy treatment, and nutritional status in patients with rectal cancer undergoing neoadjuvant chemo-radiotherapy.

## 2 Materials and methods

### 2.1 Study design

Cohort study with two groups, performed in patients with rectal adenocarcinoma in neoadjuvant treatment, from May 2021 to July 2023.

### 2.2 Study population

Adult patients with a diagnosis of adenocarcinoma of the rectum (confirmed by biopsy) in treatment with neoadjuvant chemo-radiotherapy were recruited. Patients with severe renal, cardiac, respiratory, or hepatic disease, pregnant or lactating women, or patients with an allergy or intolerance to any of the ingredients of the formula under study were excluded.

The randomization procedure was performed by the person responsible for the study’s statistical analysis, using a number table. Each patient received a participant number that assigned him/her to a specific group (PD or DA). Distribution between groups followed a 1:1 ratio.

### 2.3 Clinical study

Patient follow-up was conducted through a series of scheduled visits to assess patient status at different stages of treatment and post-treatment. The evaluations were conducted in three key visits: Visit 1 (V1, 15–20 days before starting radiotherapy), Visit 2 (V2, during radiotherapy) and Visit 3 (V3, at the end of radiotherapy). Additionally, for patients undergoing surgery, an additional evaluation was conducted 30 days post-surgery.

In V1, baseline demographic data (sex and age) were collected, along with clinical data related to the oncologic diagnosis and treatment. To determine the effect of nutritional supplementation, the following evaluations were performed in the subsequent visits:

Intestinal toxicity: Using the Common Toxicity Criteria version 5.0 of the National Cancer Institute (CTCAE v5.0), the degree of gastrointestinal toxicity associated with cancer treatment was evaluated: nausea, vomiting, abdominal pain, intestinal mucositis, diarrhea, and constipation. In addition, the following were collected: total volume radiation dose (cc), minimum, average and maximum radiated bowel (percentage and Gy), and the volume of radiated bowel (V40 < 150cc) in short cycle and long cycle.

Functionality: The scale designed by the Eastern Cooperative Oncology Group (ECOG) was used. The ECOG scale assesses the evolution of the patient’s capabilities in daily life while maintaining maximum autonomy. This data is critical when considering treatment since the therapeutic protocol and the prognosis of the disease depend on this scale. The ECOG scale is scored from 0 to 5.

Radiotherapy treatment interruptions: The percentage of patients who required interruption of treatment during follow-up was collected.

Nutritional status: Anthropometric data were collected (weight, height, calculation of the percentage of weight lost, and calculation of the body mass index), body composition (percentage of fat mass and percentage of fat-free mass), analytical data (total protein, albumin, prealbumin, C-reactive protein, cholesterol, and triglycerides), and a diagnosis of malnutrition was made following the GLIM criteria.

Surgical complications: The percentage of patients who underwent surgery who presented infectious complications, fistulas, re-interventions, re-admissions, or death 30 days after surgery was recorded. Hospital stay was also recorded.

Sensory evaluation of the nutritional supplement: A sensory evaluation was carried out in which odor, color, flavor, and perceived texture were evaluated on a semi-quantitative Likert scale (0–5). The responses were qualitatively classified as: very bad = 0, bad = 1, fair = 2, good = 3, very good = 4, or excellent = 5.

### 2.4 Nutritional treatment

Following the ESPEN recommendations for cancer patients, all patients received dietary recommendations to increase energy and nutrient intake through regular dietary intake. Moreover, patients in the intervention group that received the peptide diet were instructed to take 1–2 containers of the nutritional supplement daily (according to their nutritional needs to be covered) from day 1 of radiotherapy until the time of surgery, continuously, for a total of 12 weeks.

Formula studied (Table 1):

**TABLE 1:** Macronutrient and ingredient composition of the formula under study per 100 ml.

	PD (A)
Energy (kcal)	150 kcal
Proteins: (g/VCT%) Ingredients Glutamine: (g)	7.9 g/21 Hydrolyzed whey protein 2.05 g
Carbohydrates: (g/VCT%) Ingredients Sugars: (g)	19 g/50 Dextrin and maltodextrin 0.88 g
Fat: (g/VCT%) Ingredients MCT (%) EPA and DHA (mg)	4.8 g/29 MCT, EVOO and fish oil 70 76 mg

EVOO, extra virgin olive oil; MCT, medium chain triglycerides; PD, peptide diet; VCT%, percentage of total caloric value. A: Bi1 peptidic^®^, Adventia Pharma S.L, Spain.

∙ PD (Bi1 PEPTIDIC^®^, Adventia Pharma). Oral nutritional supplement (ONS) oligomeric, hypercaloric and hyperproteic, without fiber.

### 2.5 Ethical aspects

The study was carried out in accordance with the Helsinki declaration. The study protocol, the patient information sheet, and the informed consent form were approved by the Ethics Committee for Research with Medicines of the Hospital Universitario de Gran Canaria Doctor Negrín on May/2021 (no. 2021-189-1).

All patients were informed of the conditions of participation in the study and agreed to participate after signing the informed consent form.

### 2.6 Statistical analysis

A statistical study was carried out using the SPSS 22.0 program (IBM). Quantitative variables were evaluated for normal distribution with the Kolmogorov–Smirnov test and expressed as mean and standard distribution. A comparison between quantitative variables was performed with Student’s *t*-test.

Qualitative variables are expressed as absolute frequencies and percentages. For the comparison between variables, the chi-square test and the calculation of the relative risk (RR) with its 95% confidence interval were used. Subanalysis was performed by tumor stage, oncologic treatment (short or long), and diagnosis of malnutrition. A *p*-value of less than 0.05 was considered significant.

## 3 Results

### 3.1 Study population

**FIGURE 1:**
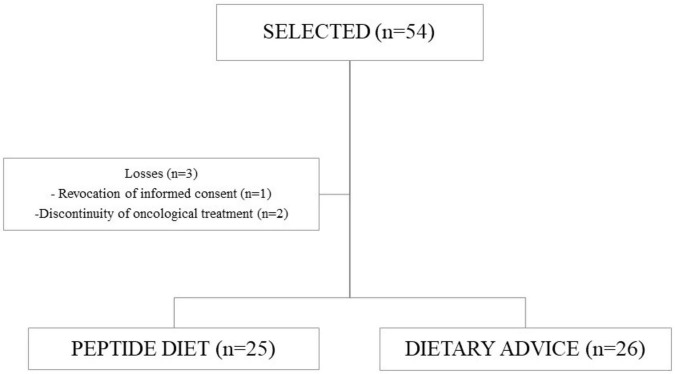
Patient flow diagram.

Fifty-four patients diagnosed with rectal adenocarcinoma under neoadjuvant treatment were initially selected. Fifty-one patients were randomized uniformly to the peptide-diet group (25 subjects) or the dietary-counseling group (26 subjects). No enrolled patients were excluded, and all completed the intervention and follow-up period (Figure 1). Table 2 presents the demographic and clinical parameters, with no differences found between intervention groups.

**TABLE 2:** Baseline descriptive demographic and oncologic variables.

	PD	DA	*p*
Age	65.80 (9.97)	63.54 (11.45)	0.456
Sex (% women)	34.6% (*n* = 9)	32.0% (*n* = 8)	0.843
Rectal cancer			0.454
Inferior	36.0% (*n* = 9)	23.1% (*n* = 6)	
Medium	40.0% (*n* = 10)	38.5% (*n* = 10)	
Superior	24.0% (*n* = 6)	38.5% (*n* = 10)	
Stadium			0.798
II	16.0% (*n* = 4)	19.2% (*n* = 5)	
III	80.0% (*n* = 20)	73.1% (*n* = 19)	
IV	4.0% (*n* = 1)	7.7% (*n* = 2)	
Radiotherapy treatment			0.676
Long (50 Gy)	52.0% (*n* = 13)	46.2% (*n* = 12)	
Short (25 Gy)	48.0% (*n* = 12)	53.8% (*n* = 14)	
Volume of irradiated intestine			
Total (cc)	1,012.37 (513.25)	1,328.70 (767.72)	0.090
Minimum volume (%)	2.36 (3.21)	5.26 (18.70)	0.448
Average volume (%)	23.49 (14.89)	29.08 (20.87)	0.278
Maximum volume (%)	93.89 (23.95)	104.14 (2.22)	0.073
Minimum volume (Gy)	0.62 (0.72)	2.40 (9.37)	0.274
Average volume (Gy)	7.82 (4.40)	10.83 (10.22)	0.505
Maximum volume (Gy)	36.24 (14.26)	37.03 (13.04)	0.348
V40 < 150 cc Long cycle (cc)	61.86 (66.66)	78.03 (59.95)	0.837
V40 < 150 cc Short cycle (cc)	58.10 (64.23)	103.32 (116.82)	0.180

cc, cubic centimeters; DA, dietary advice; Gy, gray; PD, peptide diet.

Globally, the 52.9% received chemotherapy treatment with capecitabine, 33.3% with FOLFOX (leucovorin calcium, fluorouracil, and oxaliplatin), 11.8% with XELOX (capecitabine and oxaliplatin), and 2.0% did not receive chemotherapy treatment, with no differences among the intervention groups. Regarding associated metabolic pathologies, 19.6% had diabetes mellitus, 39.2% had dyslipidemia, and 5.9% had heart disease, with no differences between intervention groups.

The supplementation pattern was 1 brik/day in 52% of the patients and 2 briks/day in 48% throughout the intervention period. Regarding supplement intake, 29.2% stopped taking the supplement, 50% took 1 brik/day, and 20.8% took 2 briks/day.

### 3.2 Clinical variables

#### 3.2.1 Intestinal toxicity

**FIGURE 2:**
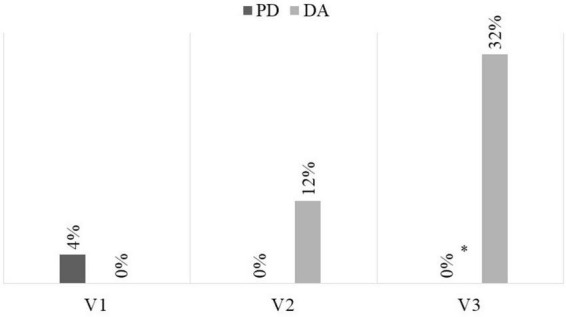
Frequency of severe diarrhea (CTCAE V5.0 ≥ 2). DA, dietary advice; PD, peptide diet; *p = 0.002.

**FIGURE 3:**
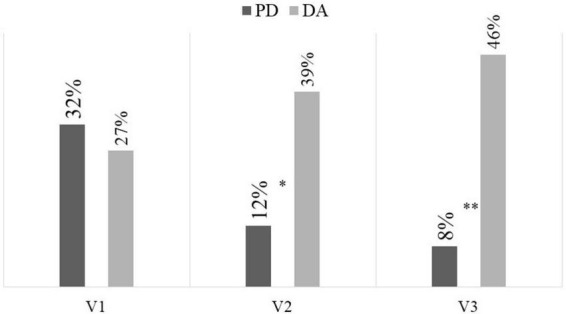
Frequency of severe mucositis (CTCAE V5.0 ≥ 2). DA, dietary advice; PD, peptide diet; *p = 0.030; **p = 0.003.

There were no differences between groups in the prevalence of nausea, vomiting, and abdominal pain at the visits performed, but there were differences in the presence of intestinal mucositis and diarrhea at the final visit, with more in the group that received DA (Table 3). When grouping the toxicity grades at ≥ 2, it was observed that toxicity related to the development of diarrhea was confirmed as more frequent in the DA group at the intermediate visit, with a RR of 0.218 (95% CI = 0.052–0.923) and at the final visit, with a RR of 0.103 (95% CI = 0.020–0.537; Figure 2). This situation was also confirmed in the development of mucositis at the final visit (Figure 3), with a RR of 0.405 (95% CI = 0.280–0.584).

**TABLE 3:** Evolution of the degree of intestinal toxicity according to CTCAE v5.0 scale.

	Grade	V1			V2			V3		
		**DA**	**PD**	***p***	**DA**	**PD**	***p***	**DA**	**PD**	***p***
Nausea	0	96.2% (*n* = 25)	100% (*n* = 25)	0.322	92% (*n* = 23)	96% (*n* = 24)	0.600	92% (*n* = 23)	96% (*n* = 24)	0.600
	1	3.8% (*n* = 1)	0% (*n* = 0)		4% (*n* = 1)	4% (*n* = 1)		4% (*n* = 1)	4% (*n* = 1)	
	2	0% (*n* = 0)	0% (*n* = 0)		0% (*n* = 0)	0% (*n* = 0)		4% (*n* = 1)	0% (*n* = 0)	
	3	0% (*n* = 0)	0% (*n* = 0)		4% (*n* = 1)	0% (*n* = 0)		0% (*n* = 0)	0% (*n* = 0)	
	4	0% (*n* = 0)	0% (*n* = 0)		0% (*n* = 0)	0% (*n* = 0)		0% (*n* = 0)	0% (*n* = 0)	
Vomiting	0	96.2% (*n* = 25)	100% (*n* = 25)	0.322	92% (*n* = 23)	100% (*n* = 25)	0.312	92% (*n* = 23)	96% (*n* = 24)	0.312
	1	3.8% (*n* = 1)	0% (*n* = 0)		0% (*n* = 0)	0% (*n* = 0)		4% (*n* = 1)	4% (*n* = 1)	
	2	0% (*n* = 0)	0% (*n* = 0)		4% (*n* = 1)	0% (*n* = 0)		0% (*n* = 0)	0% (*n* = 0)	
	3	0% (*n* = 0)	0% (*n* = 0)		4% (*n* = 1)	0% (*n* = 0)		4% (*n* = 1)	0% (*n* = 0)	
	4	0% (*n* = 0)	0% (*n* = 0)		0% (*n* = 0)	0% (*n* = 0)		0% (*n* = 0)	0% (*n* = 0)	
GI pain	0	77% (*n* = 20)	84% (*n* = 21)	0.777	92% (*n* = 23)	84% (*n* = 21)	0.384	84% (*n* = 21)	88% (*n* = 22)	0.600
	1	19.2% (*n* = 5)	12% (*n* = 3)		8% (*n* = 2)	16% (*n* = 4)		12% (*n* = 3)	12% (*n* = 3)	
	2	3.8% (*n* = 1)	4% (*n* = 1)		0% (*n* = 0)	0% (*n* = 0)		4% (*n* = 1)	0% (*n* = 0)	
	3	0% (*n* = 0)	0% (*n* = 0)		0% (*n* = 0)	0% (*n* = 0)		0% (*n* = 0)	0% (*n* = 0)	
	4	0% (*n* = 0)	0% (*n* = 0)		0% (*n* = 0)	0% (*n* = 0)		0% (*n* = 0)	0% (*n* = 0)	
Mucositis	0	100% (*n* = 26)	92% (*n* = 23)	0.339	77% (*n* = 19)	80% (*n* = 20)	0.175	56% (*n* = 13)	96% (*n* = 24)	0.009
	1	0% (*n* = 0)	4% (*n* = 1)		11.5% (*n* = 3)	0% (*n* = 0)		12% (*n* = 3)	4% (*n* = 1)	
	2	0% (*n* = 0)	4% (*n* = 1)		11.5% (*n* = 3)	0% (*n* = 0)		28% (*n* = 7)	0% (*n* = 0)	
	3	0% (*n* = 0)	0% (*n* = 0)		0% (*n* = 0)	0% (*n* = 0)		4% (*n* = 1)	0% (*n* = 0)	
	4	0% (*n* = 0)	0% (*n* = 0)		0% (*n* = 0)	0% (*n* = 0)		0% (*n* = 0)	0% (*n* = 0)	
Diarrhea	0	42.3% (*n* = 11)	48% (*n* = 12)	0.821	34.7% (*n* = 9)	62% (*n* = 15)	0.154	29.2% (*n* = 9)	88% (*n* = 22)	< 0.001
	1	30.8% (*n* = 8)	20% (*n* = 5)		26.9% (*n* = 7)	28% (*n* = 7)		25% (*n* = 6)	4% (*n* = 1)	
	2	19.2% (*n* = 5)	20% (*n* = 5)		26.9% (*n* = 7)	8% (*n* = 2)		37.5% (*n* = 9)	8% (*n* = 2)	
	3	7.7% (*n* = 2)	12% (*n* = 3)		11.5% (*n* = 3)	4% (*n* = 1)		8.3% (*n* = 2)	0% (*n* = 0)	
	4	0% (*n* = 0)	0% (*n* = 0)		0% (*n* = 0)	0% (*n* = 0)		0% (*n* = 0)	0% (*n* = 0)	
Constipation	0	80.8% (*n* = 21)	92% (*n* = 23)	0.495	92.4% (*n* = 24)	92% (*n* = 23)		92% (*n* = 23)	88% (*n* = 3)	0.637
	1	7.7% (*n* = 2)	4% (*n* = 1)		3.8% (*n* = 1)	8% (*n* = 2)		8% (*n* = 0)	12% (*n* = 3)	
	2	11.5% (*n* = 3)	4% (*n* = 1)		3.8% (*n* = 1)	0% (*n* = 0)		0% (*n* = 0)	0% (*n* = 0)	
	3	0% (*n* = 0)	0% (*n* = 0)		0% (*n* = 0)	0% (*n* = 0)		0% (*n* = 0)	0% (*n* = 0)	
	4	0% (*n* = 0)	0% (*n* = 0)		0% (*n* = 0)	0% (*n* = 0)		0% (*n* = 0)	0% (*n* = 0)	

DA, dietary advice; GI, gastrointestinal; PD, peptide diet; V1, initial visit; V2, intermediate visit; V3, final visit.

In the subanalysis performed by radiotherapy treatment (long or short), in both cases it was again confirmed that mucositis at the final visit was more prevalent in the DA group (long: 33.3 vs. 0%, *p* = 0.023; short 30.8 vs. 0%, *p* = 0.036). With respect to diarrhea, it was more frequent in the DA group at the final visit (long: 54.6 vs. 7.7%, *p* = 0.012; short: 38.5 vs. 8.3%, *p* = 0.047). In the sub-analysis performed by stage, in stage III mucositis at the final visit was more prevalent in the DA group (38.9 vs. 0%, *p* = 0.002), as was diarrhea (38.9 vs. 5%, *p* = 0.011). In the sub-analysis stratified by nutritional status, among patients with malnutrition, the prevalence of mucositis at the final visit was significantly higher in the DA group (30 vs. 0%, *p* = 0.024). Additionally, the incidence of diarrhea was greater in the DA group at both the intermediate visit (45.5 vs. 6.7%, *p* = 0.020) and the final visit (50 vs. 6.7%, *p* = 0.013). Among patients with adequate nutritional status, no significant differences were observed in the incidence of diarrhea. However, mucositis at the final visit remained more prevalent in the DA group (35.7 vs. 0%, *p* = 0.034).

#### 3.2.2 Interruption of treatment with radiotherapy

A lower rate of interruptions was observed in the group treated with PD (0%) than in the DA (11.5%), although it did not reach statistical significance (*p* = 0.070). In the subanalysis by stage, stage III patients receiving DA were observed to have a higher frequency of interruptions of radiotherapy treatment (15.8 vs. 0%, *p* = 0.049). In the sub-analysis performed by malnutrition, patients with malnutrition who received DA were observed to have a higher frequency of interruptions in radiotherapy treatment (18.2 vs. 0%, *p* = 0.040).

#### 3.2.3 Functionality

No differences were observed between groups or in the evolution of functional capacity measured by ECOG during the visits (Table 4).

**TABLE 4:** Evolution of ECOG.

	V1			V2			V3		
	**DA**	**PD**	***p***	**DA**	**PD**	***p***	**DA**	**PD**	***p***
ECOG 0	91.4% (*n* = 24)	92% (*n* = 23)	0.513	91.4% (*n* = 24)	92% (*n* = 23)	0.513	88% (*n* = 22)	96% (*n* = 24)	0.492
ECOG 1	3.8% (*n* = 1)	8% (*n* = 2)		3.8% (*n* = 1)	8% (*n* = 2)		8% (*n* = 2)	4% (*n* = 1)	
ECOG 2	0% (*n* = 0)	0% (*n* = 0)		0% (*n* = 0)	0% (*n* = 0)		0% (*n* = 0)	0% (*n* = 0)	
ECOG 3	3.8% (*n* = 1)	0% (*n* = 0)		3.8% (*n* = 1)	0% (*n* = 0)		4% (*n* = 1)	0% (*n* = 0)	

DA, dietary advice; ECOG, Eastern Cooperative Oncology Group; PD, peptide diet; V1, initial visit; V2, intermediate visit; V3, final visit.

#### 3.2.4 Nutritional status

**FIGURE 4:**
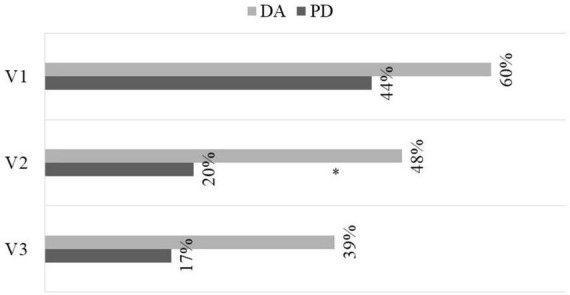
Evolution of the prevalence of malnutrition (moderate and severe) according to GLIM criteria. DA, dietary advice; PD, peptide diet; *p = 0.037.

In both groups a deterioration of nutritional status was observed, especially in the DA group (Figure 4). Regarding anthropometric and body composition parameters, no differences were detected throughout the evolution (Table 5). Regarding the analytical analysis, differences between groups were detected in the values of prealbumin in the final determination, but they were not consistent compared to the initial parameters (Table 5).

**TABLE 5:** Evolution of anthropometric, body composition, and biochemical parameters.

	V1			V3			Differences (V3-V1)		
	**DA**	**PD**	***p***	**DA**	**PD**	***P***	**DA**	**DP**	***p***
Usual weight (kg)	77.28 (12.77)	63.54 (11.45)	0.284						
Current weight (kg)	71.74 (11.19)	69.65 (9.60)	0.478	71.23 (10.50)	70.39 (9.08)	0.773	–0.15 (3.52)	–0.46 (3.03)	0.752
BMI (kg/m)^2^	25.42 (3.54)	25.17 (4.49)	0.823	28.18 (2.79)	25.56 (4.33)	0.727	–0.06 (1.21)	–0.17 (1.07)	0.759
Weight lost (%)	6.85 (5.52)	5.71 (5.86)	0.358	0.13 (5.06)	0.46 (4.91)	0.822	–0.13 (5.06)	–0.46 (4.91)	0.821
Fat mass (%)	28.54 (7.64)	25.92 (9.28)	0.278	26.86 (7.96)	26.73 (9.10)	0.952	–1.44 (2.44)	–0.29 (2.52)	0.124
Lean mass (%)	71.44 (7.63)	74.08 (9.25)	0.273	73.11 (7.93)	73.25 (9.10)	0.955	1.43 (2.43)	0.27 (2.51)	0.120
Total protein g/l	7.02 (0.43)	6.80 (0.41)	0.074	6.72 (0.65)	6.61 (0.52)	0.590	–0.29 (0.71)	–0.20 (0.50)	0.651
Albumin g/l	4.00 (0.94)	4.37 (0.36)	0.075	2.64 (1.88)	2.64 (2.21)	0.995	–1.42 (1.88)	1.60 (2.19)	0.762
Prealbumin mg/dl	24.05 (3.27)	25.90 (6.64)	0.282	22.94 (6.73)	29.45 (4.91)	0.011	0.85 (4.86)	3.70 (5.17)	0.189
CRP (mg/dl)	5.50 (5.12)	4.28 (20.39)	0.414	11.15 (12.69)	3.68 (5.30)	0.059	4.13 (9.38)	0.45 (3.15)	0.193
Cholesterol (mg/dl)	178.79 (43.57)	184.04 (43.09)	0.680	165.25 (46.21)	198.44 (58.14)	0.161	4.64 (36.69)	21.63 (32.39)	0.311
Triglycerides (mg/dl)	115.46 (52.66)	118.72 (74.67)	0.881	106.95 (29.69)	163.78 (65.99)	0.019	10.55 (32.15)	15.89 (93.29)	0.860

BMI, body mass index; CRP, C-reactive protein; DA, dietary advice; PD, peptide diet; V1, initial visit; V3, final visit.

#### 3.2.5 Surgical complications

Of the total patients, 41 underwent surgery (20 in the DA group and 21 in the PD group). No differences were observed between groups in the surgical complications evaluated (Table 6), nor in hospital stay [8.84 (10.64) days in the PD group vs. 8.60 (12.11) days in the DA group, *p* = 0.944].

**TABLE 6:** Surgical complications according to type of nutritional intervention.

	DA	PD	*P*
Infectious complications	10% (*n* = 2)	14.3% (*n* = 3)	0.598
Fistulas	0%	9.5% (*n* = 2)	0.300
Reinterventions	10% (*n* = 2)	19% (*n* = 4)	0.584
Reinstatements	5% (*n* = 1)	0%	0.477
Death	5% (*n* = 1)	0%	0.528

DA, dietary advice; PD, peptide diet.

#### 3.2.6 Sensory evaluation of the peptide diet

**FIGURE 5:**
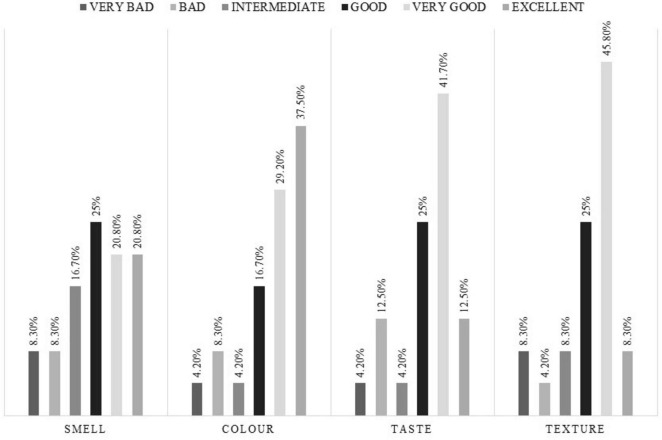
Quantitative assessment of the peptide diet under study.

Adequate acceptance of the peptide diet under study was observed (Figure 5). The mean score for odor was 3.04 (1.55), color 3.71 (1.46), flavor 3.25 (1.36), and texture 3.21 (1.35).

## 4 Discussion

Gastrointestinal toxicity, especially diarrhea and mucositis, are frequently present in patients with colorectal cancer. In our study, the comprehensive treatment of both clinical situations with a peptide enteral nutrition formula enriched with glutamine reduced the digestive toxicity associated with oncologic treatment much more than the usual clinical practice consisting of dietary advice.

The population recruited in both groups of nutritional intervention was completely homogeneous, with no difference detected between groups. In general, older patients were recruited, mainly males, with a diagnosis of rectal adenocarcinoma, mostly stage III, susceptible to receiving neoadjuvant chemo-radiotherapy treatment. Malnutrition was present in one out of two patients at the beginning of the study.

The PD diet achieved an improvement in DRTO with respect to the group that received DA exclusively. Specifically, stage III patients and patients with malnutrition presented a lower incidence of diarrhea when receiving PD compared to those who followed standard clinical practice with DA. Focusing exclusively on the peptide diet, the study by Sanz-Paris et al. (26) determined the number of stools and their consistency with the Bristol scale but did not measure the intestinal toxicity of diarrhea with the CTCAE 5.0 scale, making the results of our studies difficult to compare. The study by Peña Vivas et al. (27) did measure the presence of diarrhea with the CTCAE 5.0 scale but did not determine the degrees of toxicity, which were recorded in our study. In this case, at the final visit the prevalence of toxicity was 8% in PD and 45% in DA, values very similar to those detected by this group (5% in the PD group and 85% with a polymeric diet), also achieving in both cases a statistically significant reduction in RR in favor of the PD group (27) [RR of 0.103 (95% CI = 0.020–0.537) vs. RR of 0.059 (95% CI 0.015–0.229)].

In addition to an improvement in DRTO, an improvement in intestinal mucositis was observed in the group that received PD, an aspect of great clinical effect for the patient. In the literature reviewed, only in the study by Peña Vivas et al. (27) was this variable evaluated, and in both cases a decrease in RR in favor of PD was observed [RR of 0.405 (95% CI = 0.280–0.584) vs. RR of 0.202 (95% CI 0.102–0.399)].

Certain metabolic alterations and potential improvements may arise from the effects of the test diet (PD), likely attributed to specific bioactive components such as extra virgin olive oil (EVOO), glutamine, and the omega-3 fatty acids EPA and DHA. EVOO is rich in the phenolic compound oleocanthal, which exerts potent anti-inflammatory effects by inhibiting cyclooxygenase (COX) enzymes, specifically COX-1 and COX-2, key mediators in the biosynthesis of pro-inflammatory molecules. Attenuation of chronic inflammation, both at the intestinal and systemic levels, may significantly optimize metabolic function by reducing oxidative stress and downregulating the production of pro-inflammatory cytokines, such as IL-6 and TNF-α. This systemic anti-inflammatory effect may enhance nutrient utilization efficiency and facilitate the restoration of energy metabolism compromised by oncologic treatments (28). Glutamine, a conditionally essential amino acid, plays a pivotal role in the energy metabolism of enterocytes (intestinal epithelial cells). Under conditions of metabolic stress, such as those induced by cancer therapies, glutamine demand escalates due to its critical involvement in cellular repair and regenerative processes. Exogenous glutamine supplementation via the test diet may promote intestinal homeostasis by upregulating protein synthesis, reducing intestinal permeability, and preserving epithelial barrier integrity. These effects could enhance nutrient absorption and attenuate the protein catabolism linked to systemic inflammation and treatment-induced toxicity, thereby supporting improved nutritional status (29). EPA and DHA are implicated in mitigating metabolic dysfunctions triggered by cancer therapies and in enhancing patient immune function through modulation of inflammatory pathways and cell membrane fluidity (30). Finally, hydrolyzed proteins are characterized by an accelerated absorption profile within the gastrointestinal tract, leading to a more rapid increase in plasma amino acid levels. This faster digestion rate enables a quicker entry of amino acids into circulation, thereby augmenting the anabolic response in skeletal muscle. Additionally, hydrolyzed proteins reduce splanchnic amino acid extraction, thereby increasing peripheral availability to tissues such as muscle, and enhancing postprandial protein synthesis (31).

One of the most noteworthy results of the study is the reduction in the number of interruptions of radiotherapy treatment in the group that received PD when the onco-logic stage was III and they were malnourished. This variable was not evaluated in any of the studies reviewed, so it could shed light on the clinical effect of specific nutritional treatment with peptide formulas in patients with rectal cancer and malnutrition.

Regarding the effect on nutritional status as measured by GLIM criteria, both groups recovered during follow-up, although it was more effective in the PD group. In the case of anthropometric, body-composition, and analytical variables, there were no statistically significant differences between intervention groups. Other studies carried out with elemental and peptide diets (22–24) and peptide diets (26, 27) also showed an improvement in nutritional status at the anthropometric and analytical levels, but with greater robustness. This situation could be justified by a major methodological difference, since our study did not solely include patients at risk of malnutrition, which could have influenced the results obtained in the improvement of nutritional status.

No differences were detected in the frequency of surgical complications or hospital stay between groups. This situation could be explained by the fact that the peptide formula under study was not enriched by immunonutrients (arginine and nucleotides), nor did it contain the doses of omega-3 (EPA and DHA) that have been shown to be effective in the clinical improvement of the surgical patient (2–3 g/day) (6).

As limitations of the study, it should be noted that a dietary record was not collected, which may have limited the study of how the overall intake of the patient may have influenced their nutritional evolution. In addition, the nutritional supplementation pattern in the PD group was not homogeneous, since it was adapted to the specific nutritional needs of each patient. This situation could also be assessed as standard clinical practice since the nutritional-support regimen should always be individualized to the nutritional needs of the patient.

As strengths of the study, it should be noted that this is the first study in which the efficacy of a peptide enteral nutrition formula was evaluated during interruptions of radiotherapy treatment. This shows that specific nutritional support with a peptide formula goes beyond the simple recovery of the oncologic patient’s nutritional status and also has an effect on their clinical improvement, reducing digestive symptoms that condition their overall evolution and tolerance of oncologic treatment. This may be due to, among other possible factors, the use of partially hydrolyzed protein, the fact that the fat intake is mainly from MCT, or the fact that glutamine, the main amino acid of the enterocyte, has been supplemented.

## 5 Conclusion

In conclusion, the glutamine-enriched peptide diet had a protective effect on the development of gastrointestinal toxicity associated with antineoplastic treatment, specifically on the development of DRTO and intestinal mucositis, and reduced the interruptions of oncologic treatment in patients with colorectal cancer undergoing radiotherapy and chemotherapy.

## Data Availability

The data presented in this study are available on request from the corresponding author. The data are not public available due to ethical and privacy restrictions.
